# Case Report: Intracranial epidermoid cyst accompanied by bone hyperplasia forming a bone encapsulation

**DOI:** 10.3389/fonc.2026.1677963

**Published:** 2026-02-26

**Authors:** Liling Yang, Xinbo Sun, Hu Sun, Shuo Sun, Jianmin Yang, Jinyang Li, Longfei Shao, Yinghao Gu

**Affiliations:** 1 School of Clinical Medicine, Shandong Second Medical University, Weifang, China; 2 Department of Neurosurgery, Zibo Central Hospital, Zibo, China

**Keywords:** bone encapsulation, chronic inflammatory response, epidermoid cyst, hyperostosis, inflammatory stimulation, mechanical pressure

## Abstract

Intracranial epidermoid cysts are not true neoplasms; rather, they represent congenital benign lesions characterized by the presence of stratified squamous epithelium, commonly referred to as pearl tumors or cholesteatomas. These cysts exhibit slow growth within the cranial cavity, primarily manifesting as compressive changes and rarely inducing adjacent hyperostosis. Of particular note, the bone encapsulation phenomenon with distinct morphological features, resulting from hyperostosis, is extremely rare, with hardly any similar cases reported in the existing literature. This report provides a detailed description of an epidermoid cyst case located in the frontotemporal region. The patient, a 53-year-old female, presented with a 20-day history of dizziness, headache, accompanied by nausea and vomiting, and was subsequently admitted to our hospital for intracranial space-occupying lesion (left frontal lobe). Postoperative histopathological examination confirmed the diagnosis of epidermoid cyst with intralesional hemorrhage, inflammatory granulation tissue proliferation, and fibrosis. Radiological imaging and intraoperative observations revealed reactive hyperostosis surrounding the cyst, with partial formation of bone encapsulation. The formation of this bone hyperplasia and the bone encapsulation with a special shape is related to the synergistic action of inflammatory stimulation and mechanical pressure.

## Introduction

Intracranial epidermoid cysts are rare pseudotumors of the nervous system (1). They can occur at any age but are most common between 20 and 50 years old, accounting for over 70% of cases. It likely arises from abnormal inclusion of ectodermal remnants during neural tube formation in the third to fifth week of embryonic development (2–4). Pathology: Histologically, it is a congenital benign tumor primarily composed of stratified squamous epithelial cells, rarely exhibiting an invasive pattern. It is also known as a pearl cyst or cholesteatoma. Imaging: Intracranial epidermoid cysts typically exhibit characteristic radiographic features, including CT density approaching water and prolonged T1 and T2 relaxation times (low T1 signal, high T2 signal) (5). Intracranial epidermoid cysts are common benign congenital lesions that develop in the pontine cerebellar horn region, usually presenting as a slow-growing occupying effect. Its effect on the adjacent cranial bones is mostly characterized by compressive bone erosion or resorption, and osteogenic reactions are extremely rare. However, there are few documented cases of concomitant adjacent bone hyperplasia, the mechanism of which is not clear. More specifically, partial or complete encapsulation of the cyst by reactive hyperplastic bone, forming a bone encapsulation pattern, has hardly been reported. This unique bony response may reveal a specific pathological process in which the balance of local bone remodeling is significantly shifted toward osteogenesis under the stimulus of long-term chronic inflammation and mechanical stress. Here, we present a case of intracranial epidermoid cyst that, during surgery, was found to be associated with bone hyperplasia, bone encapsulation, and bone destruction. Pathological examination confirmed the presence of epidermoid cyst with intracystic hemorrhage, congestion, cholesterol crystal formation, as well as inflammatory granulation tissue proliferation and fibrosis.

## Case description

**Figure 1:**
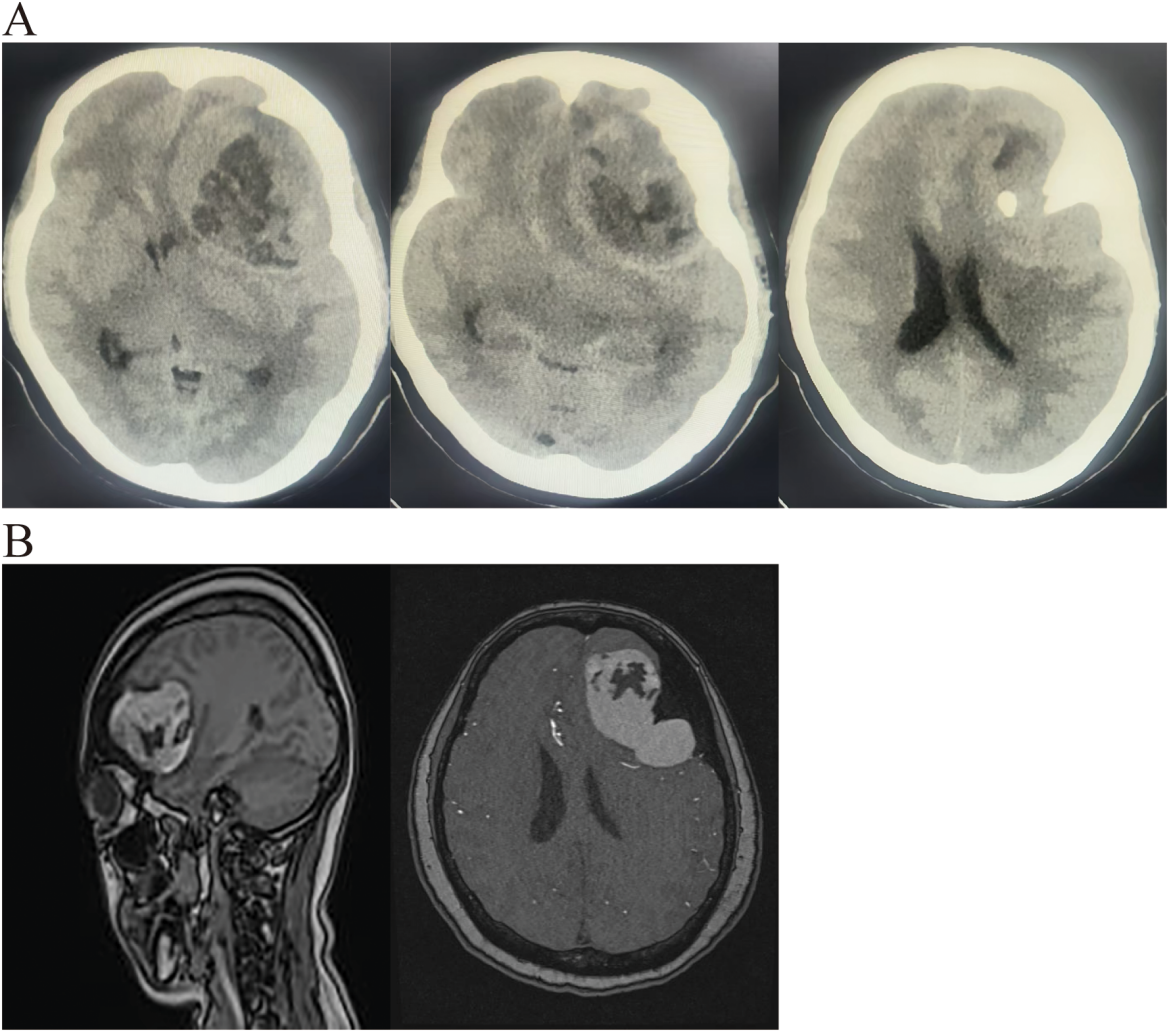
**(A)** Pre-operative CT revealed a lobulated, mixed-density lesion in the left frontotemporal lobe with associated hyperostosis of the left frontal bone. **(B)** Pre-operative MRI demonstrated a lobulated, mixed-intensity lesion in the left frontotemporal lobe consistent with an epidermoid cyst.

**Figure 2:**
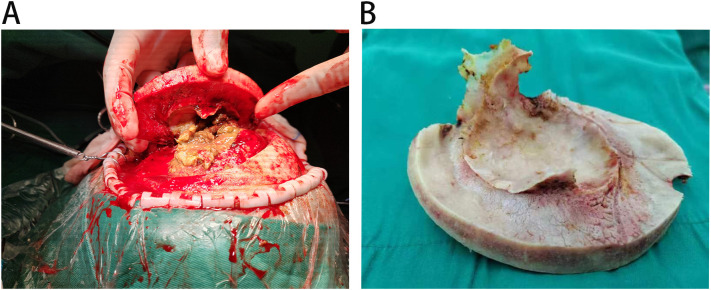
**(A)** Upon dissection, inadvertent opening of the dura and underlying cyst wall released grayish-yellow, caseous fluid. **(B)** Intraoperatively, marked focal calvarial hyperostosis and thickening were noted.

**Figure 3:**
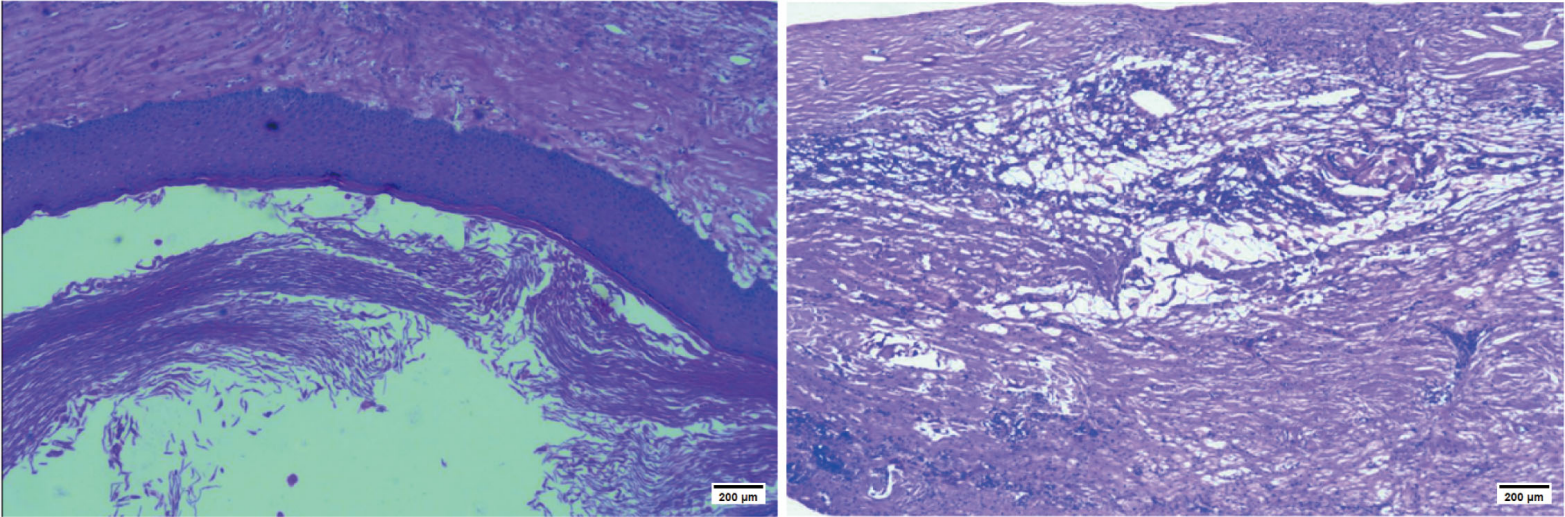
Histopathologic diagnosis (light microscopy, HE, ×10): Epidermoid cyst with intracystic hemorrhage,congestion, cholesterol-cleft formation, and associated inflammatory granulation tissue with fibrosis.

**Figure 4:**
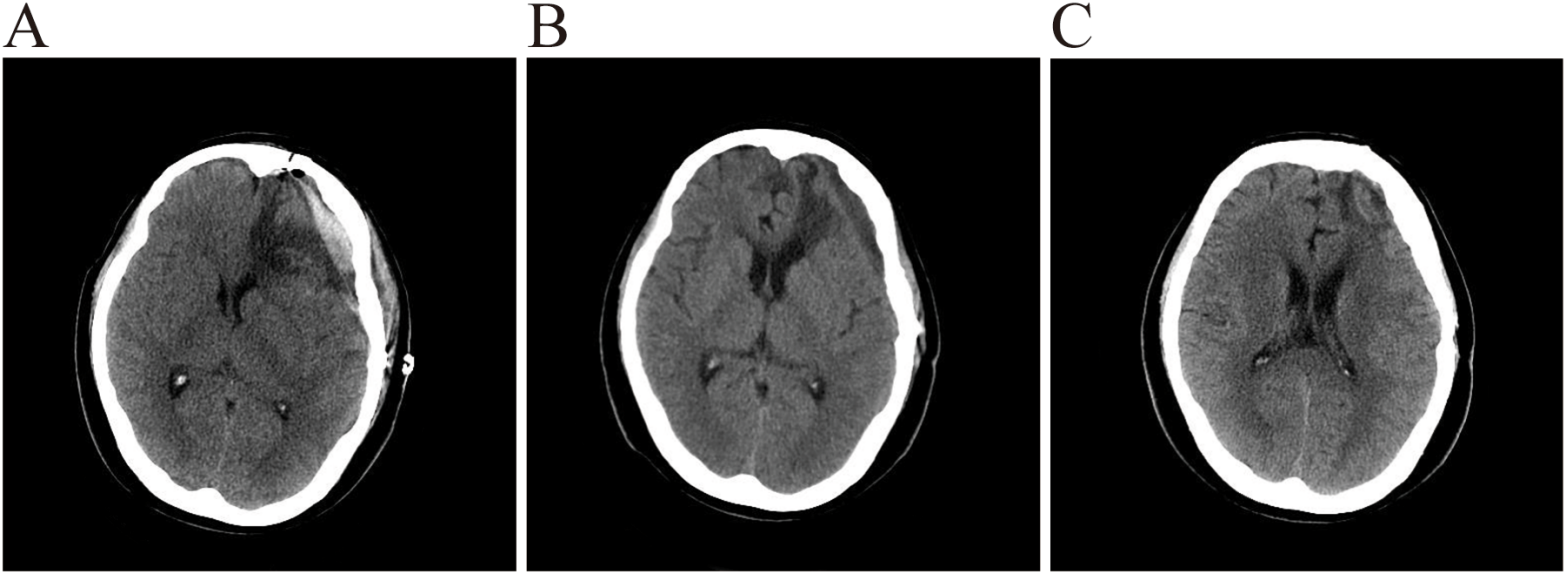
**(A)** CT demonstrates expected post-surgical changes in the left frontal region. The left frontoparietal calvarium is discontinuous; beneath the inner table, a band-like hyperdensity is seen, maximally ≈1.7 cm thick. Hyperdense streaks are noted within the left frontal lobe parenchyma. A small amount of intracranial pneumocephalus is present. Mild edema of the left frontoparietal brain parenchyma results in compression of the left lateral ventricle and a 0.5 cm rightward midline shift. Subgaleal soft-tissue swelling is evident over the left frontoparietal area. **(B)** CT again shows post-surgical changes in the left frontal region. The previously hyperdense subdural collection has evolved into a hypodense band, now ≤1.0 cm thick. Midline shift has decreased to ≈0.3 cm rightward. Subgaleal soft-tissue swelling is markedly reduced. **(C)** CT demonstrates persistent post-surgical changes. The left frontoparietal bone remains discontinuous. A thin, hypodense extra-axial collection persists beneath the inner table, measuring ≤0.4 cm at its thickest point. No significant mass effect is present; the left lateral ventricle is well visualized, and midline structures are essentially centered.

A 53-year-old female patient was admitted to the hospital with an intracranial space-occupying lesion (left frontal region). Twenty days prior, she experienced a sudden onset of headache and dizziness without any obvious precipitating factors, accompanied by nausea and vomiting, with the vomitus being gastric contents. Her physical examination and laboratory tests revealed no significant abnormalities. A cranial CT scan (**Figure 1A**) demonstrated a mass-like mixed-density shadow in the left frontal-temporal lobe. An MRI (**Figure 1B**) indicated a mixed-signal space-occupying lesion in the left frontal lobe, suggesting a benign extracerebral tumor, likely a dermoid cyst. After admission, standard neurosurgical nursing care and first-level nursing were provided. All preoperative examinations were completed to rule out surgical contraindications, and the patient and her family were informed of relevant precautions. One week after admission, the patient underwent a left frontal-temporal-parietal intracranial space-occupying lesion resection under general anesthesia. Intraoperatively, significant hyperostosis and thickening of the local skull were observed (**Figure 2B**). The bone flap was carefully separated, revealing marked hyperostosis in the central skull, which was tightly adherent to the dura mater. During the separation process, the dura mater and the underlying cyst wall were opened, and a grayish-yellow caseous fluid was discharged (**Figure 2A**). A portion of the caseous tissue was sent for intraoperative frozen pathology. The pathological diagnosis (**Figure 3**) revealed an epidermoid cyst with intracystic hemorrhage, congestion, cholesterol crystal formation, and inflammatory granulation tissue proliferation with fibrosis. Throughout the surgical procedure, the patient’s blood pressure and heart rate remained stable, and effective anesthesia was maintained. After the surgery, the patient was transferred to the neurosurgical intensive care unit. A CT scan on the first postoperative day (**Figure 4A**) showed postoperative changes in the left frontal region, with discontinuity in the left frontal-parietal bone. A band-like area of increased density was visible beneath the corresponding inner table of the skull, measuring approximately 1.7 cm at its thickest point. A streak-like area of increased density was observed in the left frontal lobe, with a small amount of pneumocephalus in the cranial cavity. The cerebral parenchyma in the left frontal-parietal lobe was slightly swollen, the left lateral ventricle was compressed, and the midline shifted to the right by approximately 0.5 cm. Subcutaneous soft tissue swelling was present in the left frontal-parietal region. On the fifth postoperative day, the patient’s mental status deteriorated. In conjunction with the postoperative CT findings, a postoperative epidural hematoma with a significant mass effect was considered, and the patient was in the peak period of cerebral edema. A follow-up CT scan was performed, and the dosage of mannitol was increased while other treatments remained unchanged. On the 16th postoperative day, the patient was in good condition, and a follow-up CT scan led to a reduction in the mannitol dosage. The surgical incision was sutured out. On the 18th postoperative day, the patient’s postoperative recovery was satisfactory. After being informed of discharge precautions, discharge procedures were completed for her. Follow-up examinations at one and a half months and one year postoperatively showed that the patient had recovered well (**Figures 4B, C**).

Written informed consent was obtained from the individual(s) for the publication of any potentially identifiable images or data included in this article.

## Discussion

Intracranial epidermoid cysts are rare, typically benign tumors with a favorable prognosis following surgical resection. Mendonça et al. noted that this condition most commonly occurs in individuals aged 30 to 40 years (6), accounting for 0.2% to 1.8% of intracranial tumors (2, 3, 7, 8), and representing 7% of cerebellopontine angle tumors (9). They predominantly arise in the cerebellopontine angle or parasphenoid regions, presenting insidiously with slow growth and typically mea suring less than 2 cm in diameter. In this case, the epidermoid cyst within the frontal bone exceeded 2cm in diameter, presenting with concurrent bone destruction and bone hyperplasia formation. As a metabolically active organ, bone undergoes continuous remodeling throughout life. Adult bone remodeling requires strict regulation to maintain bone mass homeostasis (10). The remodeling cycle comprises three sequential phases—resorption, reversal, and bone formation—involving interactions between osteoblasts and osteoclasts, and is modulated by systemic hormones and local factors (11). Bone remodeling not only adapts skeletal structure to changing mechanical demands but also repairs microdamage within the bone matrix and prevents the accumulation of old bone. Its regulatory mechanisms involve both systemic and local factors (12), which can influence the stability of the bone remodeling cycle (13). Local factors are particularly associated with the pathogenesis of skeletal fixation, inflammation, and Paget’s disease-related bone changes. The specific morphological phenomenon of a special bone encapsulation with associated bone hyperplasia in epidermoid cysts is relatively rare. Most reports involve only bone erosion or calcification, with almost no documentation of intracranial epidermoid cysts associated with bone hyperplasia. Pathological factors affecting bone remodeling include metabolic imbalance, mechanical stress, tumors, and inflammation. Therefore, its mechanism of action may be related to the combined effects of factors such as inflammatory stimulation, tumor growth characteristics, and mechanical pressure.

Daisuke Noda, Che-Kuang Lin, and colleagues propose that bone hyperplasia arises from prolonged nonspecific inflammatory responses, often associated with chronic inflammation (13, 14). For instance, persistent inflammation near the periosteum can induce reactive bone formation, the mechanism confirmed in multiple chronic inflammatory bone disorders (15). Chronic nonbacterial osteomyelitis (CNO), an autoimmune disorder considered a manifestation of SAPHO (synovitis, acne, pustulosis, osteophytes and osteitis) syndrome, illustrates this process: the acute phase involves extensive inflammatory cell infiltration leading to medullary cavity expansion; the subacute phase features the formation of reactive woven bone; and the chronic phase is characterized by medullary fibrosis with surrounding bone sclerosis (16). Similarly, Caffey disease (infantile cortical hyperostosis), a self-limiting disorder, progresses through acute periosteal inflammation, subacute subperiosteal new bone formation, and chronic inflammatory resolution with bone remodeling (17). Both conditions demonstrate that chronic inflammation can induce skeletal thickening and morphological changes (18). Clinical evidence further supports inflammation’s pivotal role in bone hyperplasia. Pushker et al.’s retrospective analysis of 280 orbital or periorbital dermoid/epidermoid cysts revealed inflammatory responses in 25% of cases (19). Elkhaary et al.’s study of 27 orbital exenteration patients found orbital osteoid bone hyperplasia in 63.0% of cases, where immediate coverage of the orbital socket with a muscle flap during surgery reduced the risk by 87.0%. This suggests that delayed healing of inflammatory granulation tissue leading to chronic osteitis is a primary mechanism of postoperative bone hyperplasia (20), aligning with findings from other cases (21). Behshad et al. also observed that prolonged inflammatory stimulation leads to reactive proliferation of the periosteum and bone (22). For example, in ankylosing spondylitis, autoimmune inflammation triggers endochondral ossification through an “inflammation-osteogenesis axis,” resulting in heterotopic ossification (23). M2 macrophages can directly promote ectopic ossification by secreting osteogenic factors such as TGF-β and BMP (24). Neutrophils and their extracellular traps (NETs) have also been shown to promote bone formation in skeletal muscle injury and play a role in ectopic ossification (25). Furthermore, IL-1β mediates interactions between neutrophils and macrophages via NETs, coordinating the transition from inflammatory response tobone formation (26). These studies indicate that both chronic and acute inflammatory responses can drive bone hyperplasia through multiple cytokines and signaling pathways. Pathological examination of this case revealed a cyst accompanied by intra-cystic hemorrhage, cholesterol crystals, and proliferative inflammatory granulation tissue with fibrosis. This suggests that the cyst wall chronically induced inflammation, with persistent inflammatory cell infiltration and release of mediators continuously stimulating the surrounding bone. This prolonged inflammatory stimulation ultimately led to bone hyperplasia through the activation of osteoblasts and promotion of bone matrix deposition.

The mechanical pressure exerted by the cyst itself represents another significant predisposing factor for hyperostosis. As an epidermoid cyst grows, it continuously produces substances such as keratin and cholesterol, leading to gradual enlargement and exerting persistent mechanical compression on the surrounding skull. Although the skull is relatively rigid, prolonged compression can induce localized bone resorption, proliferation, and destruction, potentially leading to skull thickening, thinning, or even defect formation (27). Such stress alters local bone metabolism, resulting in both bone hyperplasia and destruction (28). These cysts can reach substantial sizes, potentially causing osteolytic destruction mimicking malignant tumors; however, being benign, their clinical impact depends more on their size and growth rate (29–31). For instance, Ha et al., 2019 reported a giant epidermoid cyst with cranial bone erosion (32), and a 2023 report documented a case of cystic penetration through the skull (27). Currently reported bone lesions associated with epidermoid cysts primarily include cranial bone erosion, calcification, and atrophy (29, 32–37). However, reports of a unique bone encapsulation resulting from reactive bone hyperplasia caused by an epidermoid cyst are relatively scarce. So this article mainly introduces its aspects related to bone hyperplasia. As the cyst gradually enlarges, it exerts continuous compression on the surrounding brain tissue and skull. This mechanical pressure may lead to alterations in local blood flow within the skull, thereby affecting normal bone metabolism and promoting bone hyperplasia. Analogous to the physiological state where mechanical loading influences bone metabolism through intraosseous fluid flow (38). the pathological compression from the cyst may alter local bone metabolism via a similar mechanism. Under normal circumstances, mechanical loading can indirectly promote bone formation by downregulating the expression of Sost in osteocytes and activating the Wnt/β-catenin signaling pathway (39). In pathological states, however, abnormal mechanical stress may disturb local hemodynamics and cytokine release, leading to imbalanced bone metabolism and triggering reactive bone hyperplasia (40). This case presents concurrent bone hyperplasia and bone destruction, with the morphological pattern of bone hyperplasia being modulated by the epidermoid cyst to form a bone encapsulation encasing the cyst. We propose that this bone hyperplasia formation is associated with imbalanced bone metabolism induced by mechanical pressure generated under the pathological state of the cyst.

Although epidermoid cysts are typically benign and respond well to surgery, malignant transformation is rare. Since the initial description by Ernst-Heidelberg in 1912, systematic reviews have identified potential mechanisms for malignant transformation, including cyst rupture and repeated irritation, chronic inflammation following subtotal resection, and recurrent meningitis (41, 42). Some historical perspectives have suggested that bone hyperplasia can constitute part of a tumor process, with tumor cells invading the bone’s Haversian system, and evidence links meningioma-associated bone hyperplasia to tumor aggressiveness (43–47). However, the pathological findings in this case demonstrate intra-cystic hemorrhage, proliferative fibrous inflammatory granulation tissue, and benign histology. Therefore, the likelihood of tumor invasiveness causing the observed bone hyperplasia is considered low.

A single factor may not be sufficient to account for the formation of such a unique bone encapsulation morphology in this patient. In this case, inflammation and mechanical pressure act independently yet synergistically, jointly promoting the development of bone encapsulation. On one hand, local inflammation releases inflammatory cytokines that activate osteogenic signaling pathways. On the other hand, the continuous mechanical pressure generated by the cyst disrupts the local bone metabolic homeostasis. The synergistic interaction between these two factors ultimately gives rise to this rare condition of encapsulated bone hyperplasia. Given the rarity of such presentations and the inherent limitations of a single-case report, future studies should aim to collect patient data from multiple centers. Currently, the sample size of epidermoid cysts associated with hyperostosis remains limited. Expanding this sample size through multicenter collaboration is essential to conduct in-depth investigations into the relationship between intracranial epidermoid cysts and contributing factors such as inflammatory stimulation and mechanical stress. A deeper exploration of the underlying pathogenesis represents an important direction for future research in this field.

## Conclusion

Intracranial epidermoid cyst is a relatively special neurological disorder, and the occurrence of bone hyperplasia accompanied by the formation of a unique enveloping structure is extremely rare. This rare phenomenon may be associated with inflammatory stimulation and mechanical pressure. The cyst wall of an intracranial epidermoid cyst may trigger a chronic inflammatory response for various reasons. The continuous infiltration of inflammatory cells and the release of inflammatory mediators constantly stimulate the surrounding bone structures. Such prolonged inflammatory stimulation can lead to alterations in bone metabolism, thereby promoting the occurrence of bone hyperplasia. Secondly, the mechanical pressure exerted by the cyst itself cannot be overlooked. As the cyst gradually enlarges, it exerts sustained compression on the surrounding brain tissue and skull. This mechanical pressure may cause changes in local cranial blood flow, which in turn affects normal bone metabolism and leads to bone hyperplasia. The growth direction and velocity of the epidermoid cyst may influence its interaction with the skull, thereby increasing the risk of bone hyperplasia. Up to now, cases of epidermoid cyst accompanied by encapsulated bone hyperplasia have been extremely rare, which may be related to the interaction of the aforementioned factors.

## Data Availability

The raw data supporting the conclusions of this article will be made available by the authors, without undue reservation.
